# Death While under the Influence of Chloroform

**Published:** 1852-08

**Authors:** 


*Death while under the influence of Chloroform.*—The following are the particulars, as learned from various and direct sources, of a melancholy case which recently occurred at Hooksett, in this state:

A girl, of about fifteen years of age, had a tumor upon the thigh, which was examined by Dr. Timothy Haynes, of this town, and its removal was advised and strongly urged. After some time the patient consented that it should be done, and a day was fixed for the operation. At the appointed hour Dr. H. arrived, attended by his student; but the patient was so much terrified in the prospect of the operation, and of taking chloroform, that she ran away and hid. After some time she was found, and with a good deal of force was brought to the house and the room, where the operation was to be performed. She entreated to be allowed to go, but still more, that she might not be obliged to inhale the ether; saying that she would bear the pain of the operation, but she knew she should die if they made her breathe it. The doctor, however, insisted upon her taking it, and she was held, and concentrated chloric ether was administered by her uncle, not a physician. An unusually large amount was required before she ceased struggling violently, but finally the operation was commenced, and almost at the same time the patient was found to be exceedingly prostrated. The tumor was removed, and the doctor exerted himself to revive his patient, but in vain; she died in a very few minutes.

We regret that we cannot give the *precise* quantity of ether used and the *precise* time that elapsed between the commencement of the inhalation and the death of the patient,—but only one medical man was present, attended by a student, not at all advanced in his studies, and he proposes to "live down," that is, wait till people forget the case, not to report it. The impression of friends in such minutiæ, is perhaps, not perfectly reliable.

In view of these facts, shall we attribute the fatal result to the anæsthetic agent? We say at once, no. The patient was extremely terrified at the idea of the operation, but she was far more so at the thought of being rendered insensible. She entreated to be allowed to suffer the pain, reaffirming that she *knew* she should die if she breathed the ether. Under such circumstances, would she not have died if she had inhaled the vapor of water, believing it to be ether? Our own impression is that she would, and fright was in fact the cause of her death.

The errors in this case were, first, that the operator insisted upon the inhalation, or even *consented* to it. In fact, under such circumstances almost any man would have deferred the operation to a subsequent day, (there being no immediate danger to life from the tumor,) and then proceeded without placing the patient in a state of anæsthesia.

Second, that he allowed it to be administered by an unprofessional person, not at all acquainted with its use. An agent so powerful should be used with the greatest caution and skill, and no one should operate without placing the patient under the charge of a reliable physician, so far as this is concerned. The operator should be at liberty to devote all his attention to the operation, and not be distracted by watching the influence of the anæsthetic.

We regret that by this case discredit has been cast upon a most useful agent, and difficulties thrown in the way of its use. The following sentence from Professor Gilman's preface to Beck's Materia Medica, exactly expresses our views of the use of anæsthetics:

"Used with constant care, watched with unceasing vigilance, they are safe and most beneficent agents;—used rashly and thoughtlessly, they are so dangerous, so almost certainly fatal to life, that such use of them involves, in my judgment, an amount of moral guilt little short of that which attaches to manslaughter."—*New Hampshire Journal of Medicine.*

